# The Beneficial Effects of Guanidinoacetic Acid as a Functional Feed Additive: A Possible Approach for Poultry Production

**DOI:** 10.3390/vetsci13010046

**Published:** 2026-01-04

**Authors:** Shaaban S. Elnesr, Mohamed Shehab-El-Deen

**Affiliations:** 1 Department of Poultry Production, Faculty of Agriculture, Fayoum University, Fayoum 63514, Egypt; 2 Department of Animal and Poultry Production, College of Agriculture and Food, Qassim University, Buraydah 52571, Al-Qassim, Saudi Arabia; m.shehabeldeen@qu.edu.sa

**Keywords:** poultry, health, dietary supplements, antioxidant, performance

## Abstract

Functional feed additives offer a viable strategy for producing sustainable and healthful poultry. Guanidinoacetic acid (GAA), a non-antibiotic growth stimulant, has attracted significant interest from both investors in the poultry sector and researchers due to its distinct biological properties and multiple potential applications. GAA facilitates creatine synthesis, accelerates metabolism, and boosts poultry growth. Consequently, GAA can be considered a safe and beneficial creatine substitute, as it is the sole natural precursor of creatine. GAA meets the livestock industry’s demand for safe and effective therapies because it is non-toxic, readily degradable, and leaves no residues. Additionally, GAA is more stable and economical than creatine, making it a superior feed additive. In broiler chicks, GAA can replace arginine in practical diets containing either adequate or deficient levels of arginine. Supplementation with GAA offers promising opportunities to optimize broiler production and general health by promoting energy metabolism and protein synthesis. Commercially available feed-grade GAA has a high potential for inclusion in broiler diets. Supplementing broiler chickens with GAA may be an effective approach to improve performance parameters such as body weight and feed conversion ratio. In conclusion, dietary GAA supplementation (approximately 0.6–1.2 g/kg of diet, depending on desired impacts) can improve the productive performance of poultry. This review updates current knowledge on the impacts of GAA on productive and reproductive performance, egg quality, digestibility, antioxidant indices, and gut health in poultry.

## Simple Summary

This review updates our understanding of the impact of guanidinoacetic acid (GAA) on the productive and reproductive performance, egg quality, digestibility, antioxidant indices, and gut health in poultry. GAA is a naturally occurring amino acid derivative that serves as a direct precursor to creatine. GAA can promote energy metabolism and protein synthesis. GAA (at approximately 0.6–1.2 g/kg diet) has demonstrated positive effects on the productive and reproductive performance, egg quality, digestibility, antioxidant indices and gut health in poultry. GAA supplementation offers promising opportunities to optimize poultry production and overall health.

## 1. Introduction

Guanidinoacetic acid (GAA), known as guanidinoacetate or glycocyamine, is an amino acid derivative and an endogenous compound found in body tissues [1]. GAA serves as the essential precursor for creatine, which is widely distributed in nerve and muscle tissues [2]. GAA plays a critical role in cellular energy metabolism. GAA has attracted interest from the feed industry as a precursor to creatine due to its excellent industrial stability [3]. As a feed supplement, GAA functions as a direct metabolic precursor in creatine synthesis, helping to restore adequate cellular ATP levels and maintain overall energy balance in poultry. Feed additives that enhance energy utilization and naturally promote muscle growth are widely accepted in poultry nutrition. Guanidineacetic acid can improve poultry production and overall health by enhancing energy metabolism and protein synthesis [4]. GAA has increasingly been recognized as a viable alternative to antibiotics; for example, studies have shown that using GAA-enriched feed instead of antibiotics improves production outcomes in broiler chickens [5].

Dietary supplements containing GAA can conserve arginine in the body and further enhance growth performance by influencing arginine utilization and metabolism [6]. Furthermore, GAA is a potent precursor of creatine, which boosts muscle energy metabolism and reduces the breakdown of proteins, fats, and carbohydrates for energy, thereby accelerating animal growth and improving feed efficiency [7]. GAA supplementation may be essential for maintaining energy homeostasis in broiler chickens, especially those fed creatine-deficient diets composed exclusively of vegetable ingredients [8,9]. Including GAA in the diets of fast-growing broilers can help meet their creatine requirements. Since poultry consuming vegetable-based diets do not receive dietary creatine, they may require increased amounts of glycine and arginine to support endogenous creatine synthesis [10].

Diets supplemented with GAA may benefit broiler chickens, particularly those with rapid growth potential, due to their high creatine requirements for muscle development [8,11]. GAA increases the synthesis of phosphocreatine, which provides energy to metabolically active tissues such as the brain, gonads, and muscles. This process can improve nutrient distribution and boost productivity [8]. In broilers, GAA has been shown to improve intestinal morphology and mucosal barrier function [12,13]. Ahmadipour et al. [14] reported that adding GAA to broiler diets may be a useful approach to enhance gut health and growth performance. GAA reduces the metabolic demand for glycine and arginine in creatine synthesis, thereby making more arginine available for critical physiological functions and muscle growth [15,16]. Additionally, GAA exhibits antioxidant properties that protect muscle cells from oxidative stress and promote overall broiler health [17]. According to Li et al. [18], dietary GAA supplementation positively influences broiler growth, biochemical markers, and antioxidant capacity. GAA can serve as a potential substitute for arginine in young chicks, as demonstrated by improved feed efficiency and growth in broilers fed an arginine-deficient diet with GAA supplementation (1.2 g/kg) [9]. Zhao et al. [5] exhibited that growth performance significantly improved in birds fed diets supplemented with GAA (0.6 g/kg). Conversely, Li et al. [19] noted no significant improvement in broiler growth when GAA (0.6 g/kg) was added to the feed. This variability in results may be attributed to factors such as breed, age, and differential responses to GAA supplementation. Therefore, this review aims to assess the overall effects of dietary GAA supplementation on growth performance, egg quality, nutrient digestibility, antioxidant indices, and gut health to promote poultry production.

## 2. Comprehensive Methodology for Review

Relevant literature was retrieved from PubMed, Scopus, and Google Scholar using keywords such as ‘guanidinoacetic acid’, ‘functional feed additive’, ‘poultry production’, and ‘gut health’. Studies published between 2000 and 2025 were included based on their relevance to veterinary applications and phytochemical analysis. Relevant sources were selected by verifying that the studies focused on the health benefits of GAA and its use as a functional supplement in poultry nutrition.

## 3. Guanidinoacetic Acid and Its Effects on Metabolic Processes, Energy Utilization, and Nutrient Digestibility

**Figure 1:**
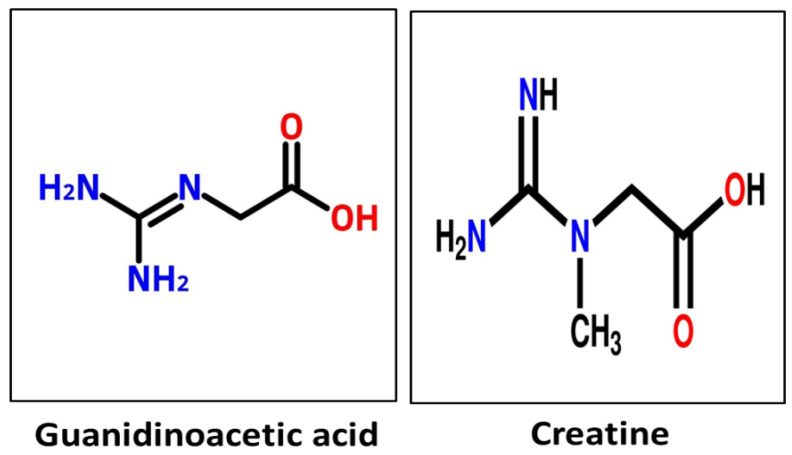
Molecular structure of guanidinoacetic acid and creatine.

Guanidinoacetic acid, commonly known as guanidinoacetate or glycocyamine, is chemically named N-[aminoiminomethyl]-glycine (chemical formula: C_3_H_7_N_3_O_2_). Figure 1 shows the molecular structure of guanidinoacetic acid and creatine. Commercially, GAA is available as a crystallized product (white crystalline powder). GAA serves as a direct precursor of creatine [2]. GAA is synthesized from the amino acids arginine and glycine in the kidney or absorbed from the gut and converted to creatine in the liver [14]. Table 1 presents the tissue involvement summary. Creatine, in its phosphorylated form, is essential for muscles as a high-energy transporter. GAA also significantly reduces the requirement for arginine [2]. The conversion of GAA to creatine requires methyl groups and produces homocysteine (Hcy), which can accept a methyl group to regenerate methionine [20]. Arginine (Arg) is converted to GAA, which is the direct precursor to creatine (Cr) and phosphocreatine (PCr), two crucial compounds for maintaining muscular energy homeostasis [21].

Nowadays, broilers are mostly fed vegetable-based diets, and exogenous creatine is not readily available. GAA, a precursor of creatine, is often used as a substitute due to its high bioavailability and notable stability [9]. Including GAA in the diet provides essential amino acids, which can be utilized in protein synthesis. The risk of creatine deficiency has increased as the consumption of animal products in diets has declined. Unlike animal proteins, creatine is absent from plant-based feedstuffs and may be lacking in all-vegetable diets. GAA has drawn a lot of interest because of its high bioavailability and notable stability. The primary function of creatine is to maximize energy efficiency across various physiological processes. The body’s effective conversion of creatine is crucial to fulfilling this role [22,23]. Endogenous creatine production may be insufficient to meet the demands of commercially farmed chickens exhibiting increased productivity or accelerated growth [24,25].

The use of GAA has shown promising results in increasing the levels of two essential compounds, creatine and arginine, which are crucial for mitigating the negative influences of free radicals and enhancing the body’s defenses against oxidative stress [5]. Also, Ostojic [26] recognized that GAA may exhibit both pro-oxidant and antioxidant properties and proposed several physiological functions of supplemental GAA beyond muscle creatine loading, including insulin sensitization and stimulation, γ-aminobutyric acid antagonism, and neuromodulation. According to Boroumandnia et al. [27], GAA may provide some protection against lactic acidemia in birds through its direct or indirect effects on the cardiovascular system. The enzyme activity of arginine/glycine amidinotransferase (AGAT) is crucial for creatine production, as AGAT catalyzes the rate-limiting and most tightly regulated step in creatine synthesis [10,28]. Table 2 illustrates the creatine biosynthesis pathway from GAA.

**Figure 2:**
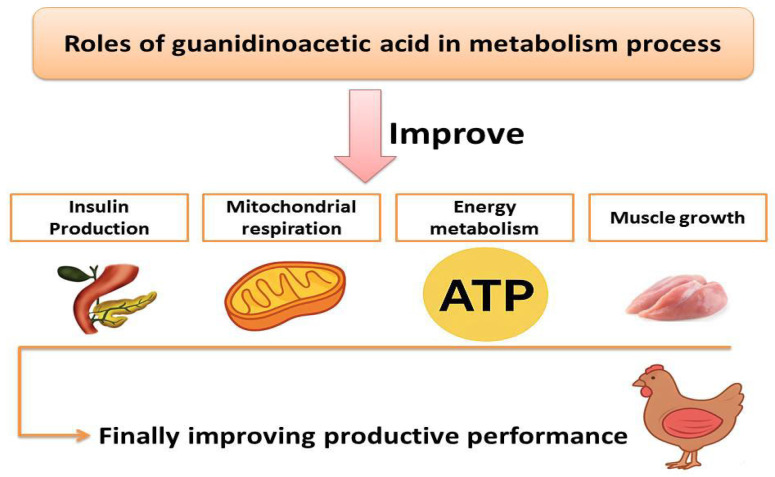
An overview of the roles of guanidinoacetic acid in metabolism process.

One of the primary regulatory enzymes in methyl group metabolism, glycine N-methyltransferase (GNMT) activity, may be positively impacted by guanidinoacetate’s direct stimulation of insulin production. GAA is necessary for energy metabolism and muscle growth, as it serves as a precursor to creatine and facilitates the synthesis of adenosine triphosphate, the primary energy molecule in cells [10,29]. DeGroot et al. [21] revealed increases in muscle glycogen in broilers fed GAA under thermoneutral conditions. Khalil et al. [30] demonstrated that GAA (0.6 g/kg) enhanced complex chain activity and mitochondrial respiration. While supplemental GAA may offer benefits for muscle energy metabolism, Boroumandnia et al. [27] noted potential adverse effects on liver and renal function. Majdeddin et al. [31] observed that dietary GAA prompts the body to utilize readily available phosphocreatine for energy production rather than relying on oxidative phosphorylation (resulting in lower glucose) and anaerobic glycogen breakdown (resulting in lower lactate). Furthermore, reduced lactate levels may indicate increased cellular stress tolerance. An overview of GAA’s roles in metabolism process is presented in Figure 2.

According to Mousavi et al. [32], GAA reduced the calorie intake per kilogram of body weight gain (BWG) by 2.9%, thereby improving energy utilization efficiency. Tossenberger et al. [26] reported that increasing dietary GAA consumption led to a significant increase (*p* < 0.05) in fecal and renal excretion of GAA, creatine, and creatinine when dietary GAA was provided at 6.0 g/kg compared to 0.6 g/kg and the control group. Fosoul et al. [23] displayed that adding GAA (1.2 g/kg) to either an energy-reduced diet (11.93 MJ/kg starter; 12.33 MJ/kg grower) or a standard energy diet (12.56 MJ/kg starter; 12.97 MJ/kg grower) reversed the negative effects of energy reduction on BWG and feed conversion ratio (FCR). Raei et al. [33] exhibited that dietary GAA supplementation in laying Japanese quail diets had both quadratic and linear effects on the ileal digestibility of dry matter, ash, ether extract, and crude protein. The beneficial effects of GAA are likely due to its enhancement of energy metabolism in intestinal epithelial cells and increased creatine production [34]. Therefore, dietary GAA supplementation facilitates nutrient digestion in the intestinal tract. It can be concluded that the inclusion of GAA may improve the rate of energy and protein absorption and metabolism. Additionally, exogenous GAA supplementation can enhance energy retention as fat in broiler chickens [23] and improve nutritional digestibility in laying Japanese quails [33]. The inclusion of GAA improved the crude protein assimilation coefficient, thereby increasing the availability of amino acids. This likely enhanced the utilization of amino acids for protein deposition, resulting in greater muscle growth [35]. Consequently, adding GAA to the diet may enhance the efficiency of protein and energy absorption and utilization, especially in low-energy diets. When low-tannin sorghum replaced corn, the beneficial effects of GAA on nitrogen-corrected apparent metabolizable energy (AMEn) and protein digestibility were more pronounced [36]. Although GAA supplementation (0.6 and 1.2 g/kg) may not provide additional benefits in diets with adequate energy levels, it can improve protein consumption and AMEn under low-metabolizable energy conditions [4].

## 4. Effect of GAA on Blood Parameters

Wyss and Kaddurah-Daouk [10] found that approximately 1.7% of the total pool of creatine and phosphocreatine is permanently converted to creatinine daily. Córdova-Noboa et al. [37] detected that treatment of GAA increased serum creatinine and creatine levels in broiler chickens. Raei et al. [33] clarified that the increasing dietary GAA levels (0.6–1.8 g/kg) elevated serum creatinine levels compared with the control birds. According to Borges et al. [35], broilers supplemented with 0.2% GAA had increased blood creatine kinase levels. Azizollahi et al. [36] displayed that adding GAA to the diets of laying hens during later stages of their life cycle resulted in higher serum creatine and creatinine levels. Tossenberger et al. [22] indicated that serum glucose, albumin, total protein, uric acid, urea, cholesterol, and enzymes GGT, AST, and ALT stayed the same when dietary inclusion of GAA was up to 0.6%. DeGroot [38] reported that no variations in AST, creatine Kinase, or mineral (P and Ca) serum concentrations were seen that might be ascribed to GAA supplementation. In broiler chicks fed both arginine-adequate and arginine-deficient diets, DeGroot [38] found that GAA supplementation only changed the differential cell proportions in heterophils and lymphocytes, without affecting the leukocyte count (lymphocytes, heterophils, basophils, monocytes, and eosinophils). DeGroot et al. [21] found that supplementing birds with 0.12% GAA as opposed to 0.0% GAA decreased heterophil proportions by an average of 35%.

## 5. Effect of GAA on Lipid Profile

Enhanced cellular energy in the liver can alter energy metabolism and may increase the activity of cholesterol 7-alpha-hydroxylase in the liver [39]. This enzyme is the first and rate-limiting step in the bile acid synthesis, converting cholesterol to 7-alpha-hydroxycholesterol. Therefore, reduced levels of circulating cholesterol may result from increased activity of this enzyme. Rahmawati and Hanim [40] clarified that GAA supplementation can lower blood cholesterol and triglyceride levels in diets with either high or low protein content. Majdeddin et al. [31] explained that GAA had no significant effect on plasma triglycerides in heat-stressed broilers, although there were slight linear decreases in plasma cholesterol. Therefore, GAA supplementation may enhance lipid turnover by stimulating the expression of both lipogenic and lipolytic genes. This metabolic adaptation could enable laying hens to produce more eggs and larger eggs [4].

## 6. Effect of GAA on Antioxidant Indices

Guanidinoacetic acid may indirectly function as an antioxidant due to the ability of its metabolites, arginine and creatine, to scavenge free radicals [5,41]. Since the methylation of GAA to creatine consumes a significant number of methyl groups derived from methionine, it is necessary to study the impacts of GAA supplementation. Michiels et al. [8] clarified that dietary GAA supplementation increases the methylation demand. Thus, the possible antioxidant benefits of supplementing with low to moderate amounts of GAA have been noted in previous studies [42]. GAA is also a strong pro-oxidant and can produce superoxide by transferring an electron from its conjugate base. Wang et al. [43] found that ducks fed GAA (0.5 g/kg) exhibited a decrease in malondialdehyde (MDA) levels. Contrary to expectations, blood GSH and GSH-Px activity, catalase activity, GSH-Px, SOD, and GSH were increased in the liver. They concluded that the elevation of creatine levels induced by GAA supplementation may enhance the body’s antioxidant capacity to some extent.

Research on broilers using GAA as a creatine source has shown that GAA supplementation (0.6 g/kg) alters several indicators of oxidative status in breast muscle by reducing MDA and reactive oxygen species (ROS) levels while increasing total antioxidant capacity. However, it does not affect SOD and GPx enzyme activities [5]. Nasiroleslami et al. [44] demonstrated that the addition level of GAA (1.2 g/kg) in broiler diet increased liver GSH-Px activity and reduced serum MDA levels. The inclusion of GAA in broiler chicken diets significantly augmented the antioxidant enzyme activities and diminished MDA levels [42]. Boroumandnia et al. [27] revealed that adding higher levels of GAA to broiler diets increased serum antioxidant indices as demonstrated by increased GSH-Px activity in the birds receiving GAA (3 g/kg). Majdeddin et al. [31] elucidated that GAA is linked to boosted muscle energy metabolism that indirectly may promote tolerance against oxidative stress in heat-stressed broilers. It is conceivable that these antioxidant impacts are based on enhanced muscle creatine loading and are therefore indirect. Therefore, the enhancement of oxidative status may be partially responsible for the performance-enhancing impacts of dietary GAA. Alaa et al. [41] explained that GAA supplementation increased GSH-Px and catalase activities and decreased nitrite concentration without significantly affecting SOD and MDA levels. Furthermore, GAA enhanced antioxidant capacity and decreased lipid peroxidation levels in broiler chickens [18]. Consequently, GAA protects broiler chickens from oxidative stress, promoting their general well-being and maintaining regular physiological processes. Additionally, Ghasemi et al. [4] elucidated that dietary GAA addition significantly elevated TAC and decreased MDA levels, indicating a positive impact on lipid peroxidation and antioxidant capacity. They showed that hens supplemented with GAA had significantly lower TBARS values and higher DPPH activity in their egg yolks, indicating that dietary GAA strengthens birds’ antioxidant defenses against oxidative stress. These results imply that while GAA supplementation may improve bird health and productivity, appropriate dosing and continuous monitoring are essential to avoid potential adverse effects.

## 7. Effect of GAA on Immunity

Dietary GAA addition in broilers exposed to heat stress reduced total leukocyte and lymphocyte counts, the latter primarily due to a decrease in the number of T cells [12]. These findings indicate that GAA may modulate cell-mediated immune responses, especially during acute heat stress. Li et al. [19] exhibited that adding GAA (0.6 g/kg) to broiler feed positively influenced immunity under heat stress by inhibiting the corticosterone synthesis and boosting plasma IL-2, IgG, and IgM levels. According to Peng et al. [13], dietary GAA improved the jejunal immunity of chicks exposed to heat stress for seven days by increasing jejunal IgA concentrations and decreasing mRNA expression of pro-inflammatory factors in the jejunum. These findings recommended that GAA might also have anti-inflammatory effects on broilers exposed to heat stress.

## 8. Effect of GAA on Gut Microbiota

GAA does not directly affect the gut environment, despite being a known precursor to creatine and enhancing creatine synthesis. The gut microbiota showed no significant changes when GAA (0.6 g/kg) was included in the diet [5]. Li et al. [18] stated that GAA had no significant effect on the composition of microbiota in chicken intestines. Specifically, they found that adding GAA (0.4 g/kg) to the feed had no significant effect on the beta and alpha diversity of microorganisms in the cecum of broiler chickens. Therefore, although GAA is recognized for its role in creatine synthesis, it appears to have little or no impact on microbial diversity.

## 9. Effect of GAA on Intestinal Integrity

**Figure 3:**
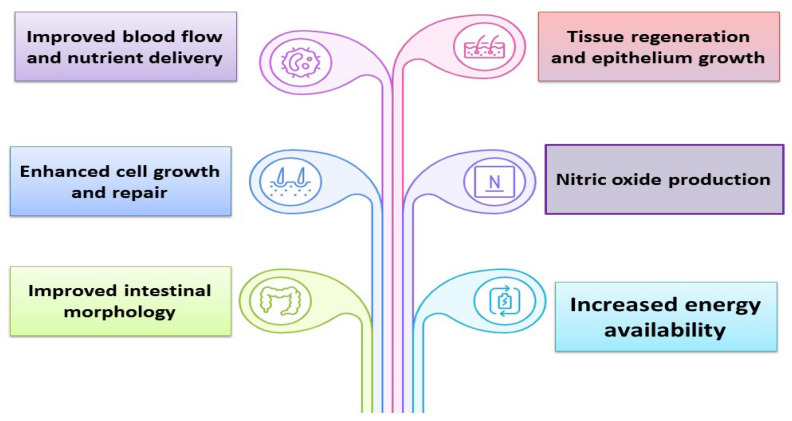
Summary of the effects of guanidinoacetic acid on gastrointestinal tract integrity in poultry.

For avian species, intestinal morphology is essential for maintaining overall health and maximizing performance. Nutraceuticals contribute to improving intestinal microstructure and development, as well as establishing a healthy intestinal microbial balance [45]. Supplementing broiler diets with GAA may be a useful strategy for enhancing growth and intestinal integrity [14]. Kodambashi Emami et al. [46] exhibited that GAA (1.2 g/kg) augmented the surface area of the jejunal villus in birds raised at cold temperatures. Ahmadipour et al. [14] illuminated that GAA addition above 0.5 g/kg significantly augmented villus height (VH), villus width (VW), and absorptive surface area in the ileum, jejunum, and duodenum. Broilers fed GAA at 0.06% or 0.12% had better surface area, VH and VW in the jejunum and duodenum [42]. Rahmawati and Hanim [40] demonstrated that the crypt depth was increased with the addition of 0.06% GAA, but the crypt depth was reduced when GAA was added at a level of 0.12%. This finding demonstrates that GAA can function as a substitute for arginine that can promote intestinal cell migration via the focal adhesion kinase and NO pathways [47]. Peng et al. [13] demonstrated that dietary GAA supplementation (0.6 g/kg) alleviated HS-induced histomorphology alterations of the small intestine and jejunal mucosal barrier dysfunction. They noted increasing the jejunal mucus thickness and goblet cell number, signifying that dietary GAA boosted the jejunal barrier function in chicks exposed to heat stress. According to Al-Abdullatif et al. [48], GAA enhanced intestinal architecture, particularly in male broilers. This study indicates that supplementing with GAA, particularly at 1.2 g/kg, may improve the growth and intestinal health of broilers, which could be beneficial. GAA supplementation significantly increased VH, CD, VW, surface area, and goblet cell count, thereby positively influencing intestinal morphology. Since arginine is a precursor for GAA synthesis, its benefits may be linked to arginine’s positive effects on intestinal health. In chicken intestinal epithelial cells, arginine has been shown to upregulate gene expression in the rapamycin signaling pathway, promoting protein synthesis and decreasing protein degradation [49]. Furthermore, arginine supports intestinal innate immunity, exhibits anti-inflammatory properties [50], and helps maintain intestinal microbiota homeostasis [51]. Thus, the improved growth performance observed in broilers fed GAA-enriched diets may be explained by the enhancements in intestinal morphology. Recently, Ghasemi et al. [4] explained that GAA supplementation positively affected the morphological parameters of the intestinal mucosa in the duodenum and jejunum. Interestingly, the best results were obtained with the highest GAA inclusion level (1.2 g/kg), especially in the duodenum. Consequently, GAA may improve intestinal morphology by increasing energy availability and promoting cell growth and repair. Additionally, GAA stimulates nitric oxide production, which improves intestinal blood flow and nutrient delivery while promoting tissue regeneration and epithelial growth [52]. Figure 3 summarizes the effects of GAA on gastrointestinal tract integrity in poultry. On the other hand, Alaa et al. [41] displayed that intestinal morphometric parameters were not significantly affected by dietary GAA inclusion. A previous study also reported no significant alterations in the gut histomorphometry in birds fed GAA (0.6 or 1.2 g/kg) [23]. Thus, the intestinal digestibility of nutrients in broilers may not be influenced by GAA supplementation. Borges et al. [35] stated that intestinal morphology was not changed by GAA inclusion. During the pre-initial rearing phase, GAA did not exhibit any activities that promote intestinal mass growth. Additionally, the inclusion of GAA (0.6 g/kg) in the diet had no effect on the development of jejunal villi [53]. These studies suggest inconsistencies in the effects of GAA on intestinal integrity. The response to GAA likely depends on various factors, including the inclusion level of GAA in the diet, genetic strain, gender, bird age, production stage, environmental conditions, and differences in the basic ingredients and components of the feed used in the research.

## 10. Effect of GAA on Reproduction

Some investigations have demonstrated that dietary GAA can improve reproductive status in poultry. GAA is a possible supplement to prevent age-related reproductive deficiencies and enhance both poultry reproduction and offspring quality [1]. Additionally, GAA supports the energy metabolism of the reproductive system. According to Sharideh et al. [54], supplementing elderly broiler breeder hens with GAA may improve sperm penetration and fertility rates by increasing ATP availability in sperm mitochondria, thereby enhancing sperm motility and fertility. Similar findings were observed by Tapeh et al. [55], who found dietary GAA at various doses improved reproductive rates and semen quality in broiler breeder roosters. Absorption and synthesis of GAA and creatine in broiler offspring were changed by adding GAA to broiler breeder diets (1.5 g/kg), which enhanced its absorption and deposition into hatching eggs [56]. Dietary GAA had a favorable impact on creatine levels in eggs and the muscular tissue of offspring in meat-type quail breeders, improving reproductive status and postnatal progeny performance [57]. The highest egg production in laying quails was achieved with 1.8 g/kg of dietary GAA supplementation [33]. According to Salah et al. [58], providing aged laying hens with GAA (1.0 or 1.5 g/kg) in their diet significantly enhanced their laying performance.

In a recent study examining laying hens in the later production stages, Pimenta et al. [59] found that feeding them GAA (0.6 and 1.2 g/kg) improved FCR. Azizollahi et al. [36] found that GAA had positive impacts on egg production, egg mass, and FCR. Supplementation of GAA in the low-ME diet, especially at 1.2 g/kg, significantly enhanced laying performance [4]. Contrary to earlier research, adding GAA to the diet may not be an effective strategy for improving the performance of laying hens [60].

Because GAA is a necessary precursor to creatine, it has a favorable effect on reproductive performance [36]. In many physiological functions, particularly in the reproductive system, creatine (as phosphocreatine) plays a crucial role as an energy carrier [10,24]. Another significant finding that sheds light on the possible mechanisms underlying the noted increases in laying performance is the rise in blood nitric oxide (NO) levels induced by the dietary GAA supplementation [36]. According to research by Manwar et al. [61], the enhanced production of NO is believed to support follicular development. This, in turn, could lead to improved ovulation and increased egg production. Uyanga et al. [62] stated that NO may play a significant role as a mediator in creating an environment conducive to optimal reproductive outcomes. This theory is supported by NO’s regulatory effects on hormone secretion and blood flow [63]. While some studies have reported no discernible advantages [59,60], others have shown enhanced laying performance after GAA addition [33,36]. The discrepancies observed across these studies may be attributed to differences in the birds’ physiological states, nutrient composition, and dietary energy levels. Additionally, variations in GAA dosage and supplementation duration likely contributed to the conflicting results reported in the literature.

## 11. Effect of GAA on Egg Quality

Because of its well-known sparing effect on arginine and methionine, GAA may indirectly increase the quality of eggs. Dietary methionine can be spared by both GAA and creatine [4]. Azizollahi et al. [36] displayed that hens supplemented with GAA showed a tendency toward greater shell thickness and a considerable improvement in shell-breaking strength. Salah et al. [58] elucidated that dietary GAA supplementation during the later production periods boosted different features of internal egg quality (yolk index, Haugh unit (HU), and albumen ratio). GAA’s function in improving the body’s efficiency in utilizing methionine explains its potential influence on egg quality. Ghasemi et al. [4] illuminated that the high GAA level (1.2 g/kg) was linked to enhanced internal egg quality, as demonstrated by a significant increase in HU. This enhancement in HU may be related to GAA’s ability to boost energy metabolism and protein synthesis. GAA supports creatine synthesis, which in turn facilitates cellular energy metabolism [26], potentially promoting better albumin protein synthesis and resulting in a higher HU [64]. Additionally, the maintenance of albumen quality may be further facilitated by a reduction in oxidative stress induced by GAA supplementation. Higher dietary GAA levels were also linked to a trend toward stronger eggshells, possibly due to its sparing influence on arginine, which is involved in calcium metabolism and absorption [65]. Previous research has demonstrated that dietary arginine supplementation can enhance eggshell thickness and promote calcium deposition [66,67]. It is well established that free radicals and ROS can compromise the integrity of cellular components involved in eggshell formation. This has led to the recognition that oxidative stress negatively affects eggshell quality [68]. According to Zhao et al. [5], the antioxidant properties of GAA may reduce oxidative stress and help maintain the eggshell integrity. GAA supplementation may increase TAC, thereby reducing oxidative damage and promoting the development of stronger eggshells.

## 12. Effect of GAA on Growth Performance

The impact of GAA supplementation on the live weight of broiler chickens has yielded conflicting results in the scientific literature. While some studies showed no significant changes [44,69], others showed positive effects [9,70]. The dosage of GAA plays a crucial role in influencing growth performance parameters. In broilers, the optimal range for GAA supplementation to improve body weight (BW) and FCR appears to be between 0.6 and 1.2 g/kg [71], while the lowest dose recommended for performance improvement is 0.6 g/kg [70]. Additionally, Michiels et al. [8] and Degroot [38] found that broiler growth performance was enhanced when GAA (0.6–1.2 g/kg) was supplemented in vegetable diets. The birds supplemented with GAA (0.6 and 1.2 g/kg) exhibited significantly elevated IGF-I circulatory levels, which may further promote muscle growth [8]. Dilger et al. [9] clarified that adding GAA (1.2 g/kg) to the diet of chicks—whose diet was based on dextrose and casein and therefore deficient in arginine—significantly enhanced their weight gain and FCR.

Mousavi et al. [32] elucidated that GAA supplementation (0.6 g/kg) decreased feed intake (23–40 d of age) and improved FCR (0–40 d and 23–40 d of age), with no significant influences on BWG. Murakami et al. [57] noted that breeder quail supplemented with GAA (0.6, 1.2, and 2.4 g/kg) had better weight gain and FCR in their progeny. Heger et al. [72] explained that GAA supplementation (0.6 g/kg) had little effect on BW or BWG but noted that FCR was a more sensitive indicator and showed an affirmative response to GAA in broiler feed. DeGroot [38] recommended that GAA supplementation improved muscle phosphagen levels in chicks fed practical diets and mitigated the growth-depressing influences of an Arg deficiency (i.e., GAA spared Arg). According to Ahmadipour et al. [14], GAA supplementation up to 1.5 g/kg improved FCR without affecting BWG or feed intake. Also, GAA supplementation significantly decreased serum uric acid concentration, which is the final byproduct of protein catabolism in birds. The reduced uric acid production in GAA-treated groups suggests improved dietary protein utilization, consistent with the observed improvement in FCR. However, they observed that a high GAA dose (2 g/kg) caused poor growth performance, although the reason for this remained unclear. According to Córdova-Noboa et al. [37], broilers fed a diet supplemented with GAA (0.6 g/kg) showed an improvement in FCR of 0.042 from day 0 to day 50 compared to birds fed the control diet. Fosoul et al. [23] elucidated that dietary GAA supplementation (1.2 g/kg) heightened the compromised growth and enhanced FCR in birds fed low-ME diets without GAA inclusion. DeGroot et al. [17] showed that feeding an arginine-deficient diet from day 1 to day 28, supplemented with GAA (1.2 g/kg), was adequate to restore BWG and FCR to those of a positive control that was adequate in Arg. He et al. [70] found that dietary GAA supplementation (0.6–1.2 g/kg) significantly enhances broiler chicken growth performance by influencing creatine metabolism and the efficiency of essential amino acid utilization. They identified 0.6 g/kg GAA as the minimum effective dose to improve performance. Regardless of the impact of the basal diet designed with normal or low protein, Amiri et al. [42] found that groups receiving GAA (0.6 and 1.2 g/kg) had a 4.52% and 5.65% decrease in FCR (0–42 days of age), respectively, in comparison to the non-supplemented groups. Additionally, average daily feed intake (ADFI) at ages 0–10, 24–42, and 0–42 days was increased by GAA supplementation. Furthermore, body weight (BW) and BWG were improved at 42 days of age. According to Faraji et al. [73], adding 1.5 g GAA/kg feed improved BWG and FCR; higher supplementation levels (2.25 g/kg) had adverse effects on these parameters. FCR has shown the most consistent impact of GAA supplementation, improving by 4.5 and 8.8 points in broilers supplemented with 0.6 and 1.2 g GAA/kg, respectively [24]. Boney et al. [74] found that the diets supplemented with GAA (0.6 g/kg) reduced FCR by 2.69% (1–21 d) and by 2.55% (36–42 d). At 21 days of age, BW was improved in comparison to the non-supplemented groups. Khalil et al. [30] found that GAA supplementation at 0.6 g/kg resulted in a 4.03% decrease in FCR relative to unsupplemented groups. Portocarero and Braun [16] clarified that GAA (0.6–1.2 g/kg) can improve FCR and increase daily BWG by 5% or more. Adding 0.6 g GAA/kg diet in broiler feed enhanced growth performance at day 10 of age [75]. Ceylan et al. [53] displayed that GAA-supplemented low-energy diets (0.6 g GAA/kg) significantly improved the performance of the birds (improving FCR and final BW by 1.66% and 1.77%, respectively). Li et al. [18] detected that the incorporation of GAA (0.4 g/kg) can significantly boost the development and growth of broiler chicks.

The combined findings of these studies recommend that GAA could be a commercially viable substance for enhancing broiler chicken growth performance. Table 3 illustrates various effects of GAA on improving growth performance in poultry. ARG, a precursor of growth-promoting polyamines, is partially formed from GAA [8,9]. The arginine-sparing effect, which makes arginine available for protein synthesis and muscle growth, was responsible for the improvements in FCR and BW observed in broilers treated with GAA [2,20]. Furthermore, creatine or its precursor, GAA, is especially important for replenishing tissue creatine stores due to the augmented muscle growth and ATP demands during the latter stages of bird life [76]. According to Tabatabaei Yazdi et al. [77], dietary supplementation with GAA may enhance the conversion of high-energy phosphates into ATP in muscles, thereby improving broiler performance. This beneficial effect is likely due to GAA’s ability to elevate muscle creatine levels [10]. The well-documented improvements in FCR can be explained by GAA supplementation reducing caloric intake per kilogram of BW, which in turn lowers FCR [32]. The advantages of GAA supplementation are more pronounced under conditions of energy restriction. GAA’s positive effects on productive performance are attributed to its role as a direct precursor of creatine, a substance essential for cellular energy metabolism and involved in numerous physiological processes, including those related to performance [24].

Enhancements in BW, BWG, and FCR show that GAA improves dietary energy utilization in broilers, even under stressful conditions [53,72]. Supplementing broiler diets with GAA (0.6 and 1.2 g/kg) during stress reduced FCR while increasing final BW and overall ADG [42]. Higher doses may spare more arginine, likely due to the sparing effect of GAA. Consequently, the dosage of GAA may determine its possible impact on broiler chicks. GAA consumption may stimulate the body to produce more phosphocreatine, providing muscles with a consistent energy supply [80]. This mechanism could explain how GAA improves growth performance in broiler chickens. However, high GAA levels (2.25 g/kg) negatively affected the growth response of broilers [73]. Excess dietary GAA significantly increases hepatic and plasma creatine concentrations in broilers [22,37]. Although dietary GAA at 2.4 and 3.0 g/kg negatively impacted growth performance, it reduced mortality in broiler chicks experiencing acute lactic acidosis [27].

Mousavi et al. [32] noticed that supplementing the broiler diet with GAA (0.6 g/kg) from days 1 to 40 increased feed intake but did not affect the birds’ weight gain. This response may result from a negative impact of GAA on ADFI or improved energy utilization in chicks fed GAA-enriched diets [72]. Tossenberger et al. [22] clarified that supplementing the diet with GAA (0.6 g/kg) did not increase growth performance because of reduced feed intake and BWG. Feed conversion ratios ranged from 1.48 to 1.49 across all treatments and were unaffected. Majdeddin et al. [81] stated that supplementation of GAA in broiler diets with varying nutrient densities decreased ADFI and FCR during the finishing period, while having no effect on performance during the growing phase. Zhang et al. [82] clarified that broilers fed diets containing GAA (1.2 g/kg) for 14 days prior to pre-slaughter showed no differences in ADG, ADFI, or feed efficiency. El-Faham [83] elucidated that feed intake was unaffected by GAA supplementation up to 0.12%. Similarly, Nasiroleslami et al. [44] reported that feed intake, BWG, and FCR were not significantly impacted by dietary supplementation with GAA (1.2 g/kg). Supplemental GAA increased FCR but had no significant effect on BW or BWG [84]. According to Cao et al. [69], GAA supplementation (0.4 g/kg) had no effect on BW, BWG, or FCR, especially when low-metabolizable-energy diets were used. These discrepancies may be attributed to species variation, GAA dosage, experiment duration, or dietary nutrient composition. The optimal GAA dose may vary depending on poultry type (broilers, layers, quail) or physiological stage (starter, grower, late lay). Table 4 summarizes the effects of GAA on poultry growth performance based on previous studies.

## 13. Strengths, Limitations, and Future Prospects

There are various advantages to using GAA in poultry feeding. Numerous independent studies consistently demonstrate improvements in feed conversion efficiency and growth performance across various experimental conditions and broiler strains. A strong scientific basis for GAA’s applications is provided by an understanding of its effects on body systems.

There are several limitations in the current study. Limited information is available on the long-term consequences or effects in some poultry species, as most studies focus on short-term effects during the growth period. Further research is needed to provide precise recommendations, since the optimal dosage may vary depending on environmental factors, bird genetics, and dietary composition. Some studies suggest that excessive GAA supplementation can have adverse consequences, underscoring the importance of proper dosage regulation.

Optimizing GAA supplementation techniques across various production systems and environmental conditions should be a primary focus of future studies. Long-term research is required to comprehensively evaluate its various effects. The efficacy of GAA can be improved by investigating its interactions with other feed additives and nutritional technologies. Studies on the effect of this compound under various stressors and disease conditions will provide valuable insights for its practical applications. Future studies should also examine the synergistic effects of combining GAA with commonly used feed additives such as betaine, methionine, and probiotics, as these are often administered together in practice. Exploring complementary pathways, including the combined provision of methyl donors and the joint enhancement of gut health, could offer important information for optimizing animal nutrition and performance. Future research will further promote the use of GAA and more precisely target its functional advantages.

## 14. Conclusions

Guanidinoacetic acid is widely recognized and utilized as a feed additive. As a natural derivative of amino acids, GAA serves as a direct precursor to creatine and has been shown to improve various physiological parameters, energy metabolism, muscle development, and growth performance in poultry. Supplementing birds with GAA may be a favorable approach to enhance their performance and metabolic efficiency. The improved performance observed in GAA-supplemented birds may result from its ability to spare arginine and glycine during metabolism. Although GAA is well known for its role in creatine synthesis, it appears to have little or no impact on gut microbiota. The magnitude and consistency of GAA’s effects vary considerably among studies and seem to be influenced by factors such as inclusion level, dietary composition, bird age and gender, production stage, and environmental conditions. Most previous studies have demonstrated that GAA supplementation at approximately 0.6–1.2 g/kg of diet positively affects productive and reproductive performance, egg quality, digestibility, antioxidant indices and gut health in poultry. Overall, GAA supplementation offers promising opportunities for optimizing poultry production and health.

## Figures and Tables

**Table 1:** Tissues Involvement Summary.

Tissues	Functions
Liver	Methylation of GAA to creatine (via guanidinoacetic acid methyltransferase)
Kidney/Pancreas	Synthesis of GAA (via Arg: Gly amidinotransferase)
Muscles/Brain	Uptake of creatine; energy buffering via phosphocreatine

**Table 2:** Creatine biosynthesis pathway from guanidinoacetic acid (GAA).

1. Formation of GAA	2. Methylation of GAA to Creatine	3. Transport and Storage
Location: Primarily occurs in the kidney and pancreas.	Location: Occurs mainly in the liver	Transport: Creatine is transported through the bloodstream to target tissues like skeletal muscle, heart and brain
Enzyme: L-arginine:glycine amidinotransferase (AGAT)	Enzyme: Guanidinoacetate N-methyltransferase (GAMT)	Storage: In these tissues, creatine is phosphorylated to phosphocreatine (PCr) by creatine kinase (CK) for use in energy buffering
Reaction: 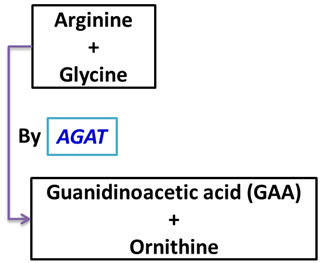	Reaction: 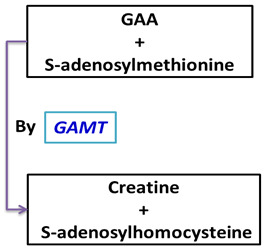	

**Table 3:** Various effects of guanidinoacetic acid (GAA) on improving growth performance in poultry.

N	Actions	References
1	Sparing Arg for use in protein synthesis and muscle size augmentation	[2,20]
2	Arg is a precursor of growth-promoting polyamines (putrescine, spermidine, and spermine) Polyamines have anabolic functions in the body, such as synthesis of DNA, RNA, and proteins, as well as the cellular uptake of amino acids	[78]
3	Regulating the phosphocreatine/creatine kinase (PCr-CK) system by GAA. The PCr-CK system plays a vital role in cellular energy metabolism through ATP regeneration	[34]
4	Improving the efficiency of energy utilization by replenishing ATP through the Cre-PCre shuttle system in addition to its Arg-sparing effect, which plays a central role in endogenous nitric oxide synthesis and maximizing growth performance	[16]
5	Redirecting amino acids (arginine and glycine) toward functions such as protein synthesis	[9,21]
6	Elevating plasma insulin-like growth factor-I	[8]
7	Improving morphology of small intestine	[79]

**Table 4:** Poultry productivity and metabolic alterations affected by dietary guanidinoacetic acid (GAA).

GAA Dose	Species	Trial Duration	Results	Reference
0.6 and 1.2 g/kg	Broiler chicks	1–42 days	Improving growth performance and FCR	[71]
0.6 and 1.2 g/kg	Broiler chicks	1–42 days	GAA can significantly enhance broiler chicken growth performance by affecting creatine metabolism and utilization efficiency of essential AA	[39]
0.6 and 1.2 g/kg	Broiler chicks	1–26 days	Improvement in feed conversion in the final period	[8]
1.2 g/kg	Broiler chicks	8–17 days	Significantly improved their weight gain and feed conversion	[9]
0.6 g/kg	Broiler chicks	0 to 40 days	Improved FCR and reduced feed intake, with no significant effects on BW gain.	[32]
0.6, 1.2, 2.4 g/kg	Quail breeders	25–29 weeks	Better weight gain and FCR in offspring	[57]
0.6 g/kg	Broiler chicks	1–35 days	Little effect on BW or BWG but improved FCR	[72]
1.5 and 2 g/kg	Broiler chicks	1–42 days	1.5 g GAA improved FCR while having no effect on BWG or feed intake. Poor growth performance was caused by the high dose of GAA (2 g/kg)	[14]
0.6 g/kg	Broiler chicks	0–50 days	Improvement in FCR of 0.042	[37]
1.2 g/kg	Broiler chicks	1–35 days	Heightened the compromised growth and enhanced the FCR of birds	[23]
0.6 and 1.2 g/kg	Broiler chicks	1–42 days	Enhancing BW, BWG, FCR and average daily feed intake	[42]
0.75, 1.5 and 2.25 g/kg	Broiler chicks	1–42 days	1.5 g GAA improved BWG and FCR; higher supplementation (2.25 g/kg) worsened these responses.	[73]
0.6 g/kg	Broiler chicks	1–42 days	Improving BW and FCR	[74]
0.6 g/kg	Broiler chicks	1–32 days	Improving FCR by 4.03%	[30]
0.6 g/kg	Broiler chicks	1–42 days	Improved feed intake, BWG and growth performance	[75]
0.6 g/kg	Broiler chicks	1–43 days	Improving final BW and FCR	[53]
0.6 and 6 g/kg	Broiler chicks	1–35 days	Feeding 0.6 g/kg GAA did not improve growth performance, whereas 6.0 g/kg GAA resulted in a reduction of feed consumption and consequently of BWG	[22]
0.6 and 1.2 g/kg	Broiler chicks	28–42 days	No differences in ADG, ADFI, or feed efficiency	[82]
0.6 and 1.2 g/kg	Qiandongnan Xiaoxiang chickens	22–24 weeks	No effect on the ADFI, ADG	[85]
1.2 g/kg	Broiler chicks	1–42 days	No effect on feed intake, body weight gain, and FCR	[44]
0.2, 0.4, 0.6 and 0.8 g/kg	Broiler chicks	1–42 days	No effect on BW, BWG, or enhanced FCR	[69]

## Data Availability

No new data were created or analyzed in this study. Data sharing is not applicable to this article.

## References

[1] Asiriwardhana M., Bertolo R.F. (2022). Guanidinoacetic acid supplementation: A narrative review of its metabolism and effects in swine and poultry. Front. Anim. Sci..

[2] Ostojic S.M. (2016). Guanidinoacetic acid as a performance-enhancing agent. Amino Acids.

[3] El-Aziz A.A., Alsenosy A.E.W., Puvača N., Tufarelli V. (2025). Guanidinoacetic acid as a functional additive in broiler nutrition: Insights into performance, carcass, antioxidant capacity, and health. World’s Poult. Sci. J..

[4] Ghasemi H.A., Azizollahi M., Lahroudi M.A., Taherpour K., Hajkhodadadi I., Akhavan-Salamat H., Rahmatnejad E. (2025). Guanidinoacetic acid in laying hen diets with varying dietary energy: Productivity, antioxidant status, yolk fatty acid profile, hepatic lipid metabolism, and gut health. Poult. Sci..

[5] Zhao W., Li J., Xing T., Zhang L., Gao F. (2021). Effects of guanidinoacetic acid and complex antioxidant supplementation on growth performance, meat quality, and antioxidant function of broiler chickens. J. Sci. Food Agric..

[6] Baker D.H. (2009). Advances in protein–amino acid nutrition of poultry. Amino Acids.

[7] Zhu Z., Gu C., Hu S., Li B., Zeng X., Yin J. (2020). Dietary guanidinoacetic acid supplementation improved carcass characteristics, meat quality and muscle fibre traits in growing–finishing gilts. J. Anim. Physiol. Anim. Nutr..

[8] Michiels J., Maertens L., Buyse J., Lemme A., Rademacher M., Dierick N.A., De Smet S. (2012). Supplementation of guanidinoacetic acid to broiler diets: Effects on performance, carcass characteristics, meat quality, and energy metabolism. Poult. Sci..

[9] Dilger R.N., Bryant-Angeloni K., Payne R.L., Lemme A., Parsons C.M. (2013). Dietary guanidino acetic acid is an efficacious replacement for arginine for young chicks. Poult. Sci..

[10] Wyss M., Kaddurah-Daouk R. (2000). Creatine and creatinine metabolism. Physiol. Rev..

[11] Nain S., Ling B., Alcorn J., Wojnarowicz C.M., Laarveld B., Olkowski A.A. (2008). Biochemical factors limiting myocardial energy in a chicken genotype selected for rapid growth. Comp. Biochem. Physiol. A Mol. Integr. Physiol..

[12] Majdeddin M., Braun U., Lemme A., Golian A., Kermanshahi H., De Smet S., Michiels J. (2020). Guanidinoacetic acid supplementation improves feed conversion in broilers subjected to heat stress associated with muscle creatine loading and arginine sparing. Poult. Sci..

[13] Peng X.Y., Xing T., Li J.L., Zhang L., Jiang Y., Gao F. (2023). Guanidinoacetic acid supplementation improves intestinal morphology, mucosal barrier function of broilers subjected to chronic heat stress. J. Anim. Sci..

[14] Ahmadipour B., Khajali F., Sharifi M.R. (2018). Effect of guanidinoacetic acid supplementation on growth performance and gut morphology in broiler chickens. Poult. Sci. J..

[15] DeGroot A.A., Braun U., Dilger R.N. (2019). Guanidinoacetic acid is efficacious in improving growth performance and muscle energy homeostasis in broiler chicks fed arginine-deficient or arginine-adequate diets. Poult. Sci..

[16] Portocarero N., Braun U. (2021). The physiological role of guanidinoacetic acid and its relationship with arginine in broiler chickens. Poult. Sci..

[17] Ncho C.M., Gupta V., Choi Y.H. (2023). Effects of dietary glutamine supplementation on heat-induced oxidative stress in broiler chickens: A systematic review and meta-analysis. Antioxidants.

[18] Li X., Chen Z., Li J. (2024). Effects of Guanidine Acetic Acid on the Growth and Slaughter Performance, Meat Quality, Antioxidant Capacity, and Cecal Microbiota of Broiler Chickens. Vet. Sci..

[19] Li X., Bian J., Xing T., Zhao L., Li J., Zhang L., Gao F. (2023). Effects of guanidinoacetic acid supplementation on growth performance, hypothalamus-pituitary-adrenal axis, and immunity of broilers challenged with chronic heat stress. Poult. Sci..

[20] Bertolo R.F., McBreairty L.E. (2013). The nutritional burden of methylation reactions. Curr. Opin. Clin. Nutr. Metab. Care.

[21] DeGroot A.A., Braun U., Dilger R.N. (2018). Efficacy of guanidinoacetic acid on growth and muscle energy metabolism in broiler chicks receiving arginine-deficient diets. Poult. Sci..

[22] Tossenberger J., Rademacher M., Németh K., Halas V., Lemme A.J.P.S. (2016). Digestibility and metabolism of dietary guanidino acetic acid fed to broilers. Poult. Sci..

[23] Fosoul S.S.A.S., Azarfar A., Gheisari A., Khosravinia H. (2018). Energy utilisation of broiler chickens in response to guanidinoacetic acid supplementation in diets with various energy contents. Br. J. Nutr..

[24] Khajali F., Lemme A., Rademacher-Heilshorn M. (2020). Guanidinoacetic acid as a feed supplement for poultry. World’s Poult. Sci. J..

[25] Majdeddin M., Golian A., Kermanshahi H., Michiels J., De Smet S. (2019). Effects of methionine and guanidinoacetic acid supplementation on performance and energy metabolites in breast muscle of male broiler chickens fed corn-soybean diets. Br. Poult. Sci..

[26] Ostojic S.M. (2015). Cellular bioenergetics of guanidinoacetic acid: The role of mitochondria. J. Bioenerg. Biomembr..

[27] Boroumandnia Z., Khosravinia H., Masouri B., Parizadian Kavan B. (2021). Effects of dietary supplementation of guanidinoacetic acid on physiological response of broiler chicken exposed to repeated lactic acid injection. Ital. J. Anim. Sci..

[28] Ostojic S.M. (2014). An alternative mechanism for guanidinoacetic acid to affect methylation cycle. Med. Hypotheses.

[29] Brosnan J.T., Brosnan M.E. (2007). Creatine: Endogenous metabolite, dietary, and therapeutic supplement. Annu. Rev. Nutr..

[30] Khalil S., Al-Sagan A.A., Abdellatif H.A., Prince A., El-Banna R. (2021). Effects of guanidinoacetic acid supplementation on zootechnical performance and some biometric indices in broilers challenged with T3-Hormone. Ital. J. Anim. Sci..

[31] Majdeddin M., Braun U., Lemme A., Golian A., Kermanshahi H., De Smet S., Michiels J. (2023). Effects of feeding guanidinoacetic acid on oxidative status and creatine metabolism in broilers subjected to chronic cyclic heat stress in the finisher phase. Poult. Sci..

[32] Mousavi S.N., Afsar A., Lotfollahian H. (2013). Effects of guanidinoacetic acid supplementation to broiler diets with varying energy contents. J. Appl. Poult. Res..

[33] Raei A., Karimi A., Sadeghi A. (2020). Performance, antioxidant status, nutrient retention and serum profile responses of laying Japanese quails to increasing addition levels of dietary guanidinoacetic acid. Ital. J. Anim. Sci..

[34] Wallimann T., Tokarska-Schlattner M., Schlattner U. (2011). The creatine kinase system and pleiotropic effects of creatine. Amino Acids.

[35] Borges K.M., Mello H.H.D.C., Café M.B., Arnhold E., Xavier H.P., de Oliveira H.F., Mascarenhas A.G. (2021). Effect of dietary inclusion of guanidinoacetic acid on broiler performance. Revista Colomb. Cienc. Pec..

[36] Azizollahi M., Ghasemi H.A., Foroudi F., Hajkhodadadi I. (2024). Effect of guanidinoacetic acid on performance, egg quality, yolk fatty acid composition, and nutrient digestibility of aged laying hens fed diets with varying substitution levels of corn with low-tannin sorghum. Poult. Sci..

[37] Córdova-Noboa H.A., Oviedo-Rondón E.O., Sarsour A.H., Barnes J., Sapcota D., López D., Braun U. (2018). Effect of guanidinoacetic acid supplementation on live performance, meat quality, pectoral myopathies and blood parameters of male broilers fed corn-based diets with or without poultry by-products. Poult. Sci..

[38] DeGroot A. (2015). Efficacy of Dietary Guanidinoacetic Acid in Broiler Chicks. Master’s Thesis.

[39] Hu X., Wang Y., Sheikhahmadi A., Li X., Buyse J., Lin H., Song Z. (2019). Effects of glucocorticoids on lipid metabolism and AMPK in broiler chickens’ liver. Comp. Biochem. Physiol. B Biochem. Mol. Biol..

[40] Rahmawati D., Hanim C. (2022). Supplementation of guanidinoacetic acid in feed with different levels of protein on intestinal histomorphology, serum biochemistry, and meat quality of broiler. J. Indones. Trop. Anim. Agric..

[41] Alaa M., Abdel Razek A.H., Tony M.A., Yassin A.M., Warda M., Awad M.A., Bawish B.M. (2024). Guanidinoacetic acid supplementation and stocking density effects on broiler performance: Behavior, biochemistry, immunity, and small intestinal histomorphology. Acta Vet. Scand..

[42] Amiri M., Ghasemi H.A., Hajkhodadadi I., Farahani A.H.K. (2019). Efficacy of guanidinoacetic acid at different dietary crude protein levels on growth performance, stress indicators, antioxidant status, and intestinal morphology in broiler chickens subjected to cyclic heat stress. Anim. Feed. Sci. Technol..

[43] Wang Y., Liu Q., Jiang F., Yuan Q., Yan R., Zhuang S. (2016). Effects of guanidinoacetic acid on performance and antioxidant capacity in Cherry Valley ducks. J. Nanjing Agric. Univ..

[44] Nasiroleslami M., Torki M., Saki A.A., Abdolmohammadi A.R. (2018). Effects of dietary guanidinoacetic acid and betaine supplementation on performance, blood biochemical parameters and antioxidant status of broilers subjected to cold stress. J. Appl. Anim. Res..

[45] Ebeid T.A., Aljabeili H.S., Al-Homidan I.H., Volek Z., Barakat H. (2023). Ramifications of heat stress on rabbit production and role of nutraceuticals in alleviating its negative impacts: An updated review. Antioxidants.

[46] Kodambashi Emami N., Golian A., Rhoads D.D., Danesh Mesgaran M. (2017). Interactive effects of temperature and dietary supplementation of arginine or guanidinoacetic acid on nutritional and physiological responses in male broiler chickens. Br. Poult. Sci..

[47] Rhoads J.M., Chen W., Gookin J., Wu G.Y., Fu Q., Blikslager A.T., Romer L.H. (2004). Arginine stimulates intestinal cell migration through a focal adhesion kinase dependent mechanism. Gut.

[48] Al-Abdullatif A.A., Azzam M.M., Samara E.M., Al-Badwi M.A., Dong X., Abdel-Moneim A.M.E. (2024). Assessing the Influence of Guanidinoacetic Acid on Growth Performance, Body Temperature, Blood Metabolites, and Intestinal Morphometry in Broilers: A Comparative Sex-Based Experiment. Animals.

[49] Yuan C., Ding Y., He Q., Azzam M.M.M., Lu J.J., Zou X.T. (2015). L-arginine upregulates the gene expression of target of rapamycin signaling pathway and stimulates protein synthesis in chicken intestinal epithelial cells. Poult. Sci..

[50] Pérez de la Lastra J.M., Curieses Andrés C.M., Andrés Juan C., Plou F.J., Pérez-Lebeña E. (2023). Hydroxytyrosol and arginine as antioxidant, anti-inflammatory and immunostimulant dietary supplements for COVID-19 and long COVID. Foods.

[51] Yang Y., Bin P., Tao S., Zhu G., Wu Z., Cheng W., Wei H. (2021). Evaluation of the mechanisms underlying amino acid and microbiota interactions in intestinal infections using germ-free animals. Infect. Microbes Dis..

[52] Ceylan N., Koca S., Adabi S.G., Kahraman N., Bhaya M.N., Bozkurt M.F. (2021). Effects of dietary energy level and guanidino acetic acid supplementation on growth performance, carcass quality and intestinal architecture of broilers. Czech J. Anim. Sci..

[53] Dao H.T., Swick R.A. (2021). New insights into arginine and arginine-sparing effects of guanidinoacetic acid and citrulline in broiler diets. World’s Poult. Sci. J..

[54] Sharideh H., Esmaeile Neia L., Zaghari M., Zhandi M., Akhlaghi A., Lotfi L. (2016). Effect of feeding guanidinoacetic acid and L-arginine on the fertility rate and sperm penetration in the perivitelline layer of aged broiler breeder hens. J. Anim. Physiol. Anim. Nutr..

[55] Tapeh R.S., Zhandi M., Zaghari M., Akhlaghi A. (2017). Effects of guanidinoacetic acid diet supplementation on semen quality and fertility of broiler breeder roosters. Theriogenology.

[56] Reicher N., Epstein T., Gravitz D., Cahaner A., Rademacher M., Braun U., Uni Z. (2020). From broiler breeder hen feed to the egg and embryo: The molecular effects of guanidinoacetate supplementation on creatine transport and synthesis. Poult. Sci..

[57] Murakami A.E., Rodrigueiro R.J.B., Santos T.C., Ospina-Rojas I.C., Rademacher M. (2014). Effects of dietary supplementation of meat-type quail breeders with guanidinoacetic acid on their reproductive parameters and progeny performance. Poult. Sci..

[58] Salah A.S., Ahmed-Farid O.A., El-Tarabany M.S. (2020). Effects of Guanidinoacetic acid supplements on laying performance, egg quality, liver nitric oxide and energy metabolism in laying hens at the late stage of production. J. Agric. Sci..

[59] Pimenta J.G., Barbosa H.J., Fraga M.G., Triginelli M.V., Costa B.T., Ferreira M.A., Lara L.J. (2023). Inclusion of guanidinoacetic acid in the diet of laying hens at late phase of feeding. Anim. Prod. Sci..

[60] Khakran G., Chamani M., Foroudi F., Sadeghi A.A., Afshar M.A. (2018). Effect of guanidine acetic acid addition to corn-soybean meal based diets on productive performance, blood biochemical parameters and reproductive hormones of laying hens. Kafkas Univ. Vet. Fak. Derg..

[61] Manwar S.J., Moudgal R.P., Sastry K.V.H., Mohan J., Tyagi J.B.S., Raina R. (2006). Role of nitric oxide in ovarian follicular development and egg production in Japanese quail (Coturnix coturnix japonica). Theriogenology.

[62] Uyanga V.A., Xin Q., Sun M., Zhao J., Wang X., Jiao H., Lin H. (2022). Research Note: Effects of dietary L-arginine on the production performance and gene expression of reproductive hormones in laying hens fed low crude protein diets. Poult. Sci..

[63] Gladwin M.T., Raat N.J., Shiva S., Dezfulian C., Hogg N., Kim-Shapiro D.B., Patel R.P. (2006). Nitrite as a vascular endocrine nitric oxide reservoir that contributes to hypoxic signaling, cytoprotection, and vasodilation. Am. J. Physiol. Heart Circ. Physiol..

[64] Vlčková J., Tůmová E., Míková K., Englmaierová M., Okrouhlá M., Chodová D. (2019). Changes in the quality of eggs during storage depending on the housing system and the age of hens. Poult. Sci..

[65] Yaman F., Acikan I., Dundar S., Simsek S., Gul M., Ozercan I.H., Sahin K. (2016). Dietary arginine silicate inositol complex increased bone healing: Histologic and histomorphometric study. Drug Des. Devel. Ther..

[66] Lieboldt M.A., Halle I., Frahm J., Schrader L., Weigend S., Preisinger R., Dänicke S. (2015). Effects of long-term graded L-arginine supply on growth development, egg laying and egg quality in four genetically diverse purebred layer lines. J. Poult. Sci..

[67] Sahin K., Orhan C., Tuzcu M., Hayirli A., Komorowski J.R., Sahin N. (2018). Effects of dietary supplementation of arginine-silicate-inositol complex on absorption and metabolism of calcium of laying hens. PLoS ONE.

[68] Ding X., Cai C., Jia R., Bai S., Zeng Q., Mao X., Wang J. (2022). Dietary resveratrol improved production performance, egg quality, and intestinal health of laying hens under oxidative stress. Poult. Sci..

[69] Cao S., He W., Qi G., Wang J., Qiu K., Ayalew H., Wu S. (2024). Inclusion of guanidinoacetic acid in a low metabolizable energy diet improves broilers growth performance by elevating energy utilization efficiency through modulation serum metabolite profile. J. Anim. Sci..

[70] He D., Yang L., Li J., Dong B., Lai W., Zhang L. (2019). Effects of guanidinoacetic acid on growth performance, creatine metabolism and plasma amino acid profile in broilers. J. Anim. Physiol. Anim. Nutr..

[71] Lemme A., Ringel J., Rostagno H.S., Redshaw M.S. Supplemental guanidino acetic acid improved feed conversion, weight gain, and breast meat yield in male and female broilers. Proceedings of the 16th European Symposium on Poultry Nutrition.

[72] Heger J., Zelenka J., Machander V., de la Cruz C., Lešták M., Hampel D. (2014). Effects of guanidinoacetic acid supplementation to broiler diets with varying energy content. Acta Univ. Agric. Silvic. Mendel. Brun..

[73] Faraji M., Karimi Dehkordi S., Zamiani Moghadam A.K., Ahmadipour B., Khajali F. (2019). Combined effects of guanidinoacetic acid, coenzyme Q10 and taurine on growth performance, gene expression and ascites mortality in broiler chickens. J. Anim. Physiol. Anim. Nutr..

[74] Boney J.W., Patterson P.H., Solis F. (2020). The effect of dietary inclusions of guanidinoacetic acid on D1-42 broiler performance and processing yields. J. Appl. Poult. Res..

[75] de Souza C., Eyng C., Viott A.M., De Avila A.S., Pacheco W.J., Junior N.R., Nunes R.V. (2021). Effect of dietary guanidinoacetic acid or nucleotides supplementation on growth performances, carcass traits, meat quality and occurrence of myopathies in broilers. Livest. Sci..

[76] Ibrahim D., El Sayed R., Abdelfattah-Hassan A., Morshedy A.M. (2019). Creatine or guanidinoacetic acid? Which is more effective at enhancing growth, tissue creatine stores, quality of meat, and genes controlling growth/myogenesis in Mulard ducks. J. Appl. Anim. Res..

[77] Tabatabaei Yazdi F., Golian A., Zarghi H., Varidi M. (2017). Effect of wheat-soy diet nutrient density and guanidine acetic acid supplementation on performance and energy metabolism in broiler chickens. Ital. J. Anim. Sci..

[78] Khajali F., Wideman R.F. (2010). Dietary arginine: Metabolic, environmental, immunological and physiological interrelationships. World’s Poult. Sci. J..

[79] Ren Q.C., Xuan J.J., Yan X.C., Hu Z.Z., Wang F. (2018). Effects of dietary supplementation of guanidino acetic acid on growth performance, thigh meat quality and development of small intestine in Partridge-Shank broilers. J. Agric. Sci..

[80] Yan Z., Yan Z., Liu S., Yin Y., Yang T., Chen Q. (2021). Regulative mechanism of guanidinoacetic acid on skeletal muscle development and its application prospects in animal husbandry: A review. Front. Nutr..

[81] Majdeddin M., Golian A., Kermanshahi H., De Smet S., Michiels J. (2018). Guanidinoacetic acid supplementation in broiler chickens fed on corn-soybean diets affects performance in the finisher period and energy metabolites in breast muscle independent of diet nutrient density. Br. Poult. Sci..

[82] Zhang L., Li J.L., Wang X.F., Zhu X.D., Gao F., Zhou G.H. (2019). Attenuating effects of guanidinoacetic acid on preslaughter transport-induced muscle energy expenditure and rapid glycolysis of broilers. Poult. Sci..

[83] El-Faham A.I., Abdallah A.G., El-Sanhoury M.H.S., Ali N.G., Abddelaziz M.A.M., Abdelhady A.Y.M., Arafa A.S.M. (2019). Effect of graded levels of guanidine acetic acid in low protein broiler diets on performance and carcass parameters. Egypt. J. Nutr. Feeds.

[84] Maynard C.J., Nelson D.S., Rochell S.J., Owens C.M. (2023). Reducing broiler breast myopathies through supplementation of guanidinoacetic acid in broiler diets. J. Appl. Poult. Res..

[85] Zhang B., Liu N., He Z., Song P., Hao M., Xie Y., Sun Z. (2021). Guanidino-acetic acid: A scarce substance in biomass that can regulate postmortem meat glycolysis of broilers subjected to pre-slaughter transportation. Front. Bioeng. Biotechnol..

